# Healthcare Facilities and Medical Tourism Across the World: A Bibliometric Analysis

**DOI:** 10.21315/mjms2024.31.2.3

**Published:** 2024-04-23

**Authors:** Abdul Latief, Maria Ulfa

**Affiliations:** 1 Master of Hospital Administration, Postgraduate Program, Universitas Muhammadiyah Yogyakarta, Yogyakarta, Indonesia; 2 RSUD Kota Yogyakarta, Yogyakarta, Indonesia; 3 School of Medicine, Faculty of Medicine and Health Sciences, Universitas Muhammadiyah Yogyakarta, Yogyakarta, Indonesia

**Keywords:** bibliometric analysis, development, healthcare facility, hospital, medical tourism

## Abstract

This study aimed to review the literature on healthcare facilities and medical tourism from a range of nations that have established medical tourism sectors and assess the effect of healthcare facilities on medical tourism. A bibliometric study of the Scopus database was carried out by using the search terms ‘(Facility AND of AND healthcare) AND TITLE-ABS-KEY (health AND tourism) AND medical tourism’ for the years 2012–2022. A qualitative evaluation of the literature was then performed to find and locate 92 articles. VOSviewer and NVivo 12 Plus were employed for data analysis. The findings indicated that the following trending subject keywords were used during the period in question: health (rate 1.97), medicine (rate 1.91), tourism (rate 1.70), care (rate 0.83), facilities (rate 0.64) and healthcare (rate 0.61). Furthermore, this research identified four distinct clusters: i) medical tourism, ii) healthcare quality, iii) healthcare system and iv) health services. The study found that healthcare facilities, as actors that have a role in the development of medical tourism, have not been sufficiently explored, even though there is evidence that they play a role in the growth of the sector. This result is in line with Heung’s argument, which makes the same point.

## Introduction

Medical tourism entails receiving medical care while traveling to another country (1). It consists of traveling across borders to receive medical treatment unavailable in the tourist’s country of origin. There are four broad categories of medical tourists: i) individuals who suffer injuries or develop medical conditions while on holiday; ii) people who visit a country with the primary aim of receiving treatment and those who decide to make use of the healthcare services of a particular nation after visiting it; iii) persons who make the journey for tourism-related purposes while receiving treatment and iv) individuals who seek treatment without any connection to tourism (2). The high costs of healthcare in developed nations and the abundance of skilled medical professionals in Asia are major contributing factors to the rapid growth of medical tourism in developing nations, including India, Malaysia, Thailand, Turkey and Singapore (3).

The literature on medical concerns frequently deals with medical tourism as a health service that can enhance international tourism. Medical tourism involves the growth of healthcare with an emphasis on tourism-related services in countries that are popular vacation destinations. The medical tourism industry is rapidly expanding, with many individuals seeking healthcare procedures such as cosmetic, dental, optical and surgical treatments that may not be available or affordable in their home countries (4). Medical tourism has emerged as a lucrative source of revenue for several developed nations (5). The effectiveness of healthcare services significantly influences medical tourism’s usefulness as a service that promotes health development. The accessibility of health services impacts the selection of medical tourism destinations. Healthcare facilities with adequate human resources, medical apparatuses and work protocols can establish medical tourism-related services. Medical tourism is one of the fastest-growing industries worldwide, and many nations are currently developing legal and operational strategies to accommodate it (6). Reduced transportation costs, increased incomes, knowledge and technology transfer, and competitive prices encourage medical travel to distant countries.

Numerous nations have begun to position medical tourism as a strategic health service development point to boost visitors’ growth. Numerous individuals opt to seek medical treatments abroad for various reasons, including the ability to receive prompt and top-notch medical care, an opportunity to discover new countries or exotic locations, greater flexibility in scheduling medical services, and the likelihood of facing steep health insurance premiums in their home nations (7, 8). Providing high-quality healthcare facilities is believed to also contribute to expanding the sector. Medical tourism as a health service that can improve international tourism is frequently an issue of concern. The development of healthcare with an emphasis on services that assist tourism in countries that are major vacation destinations is known as medical tourism (9). The efficiency of healthcare services considerably impacts medical tourism’s value as a sector that encourages the development of well-being. One factor influencing the choice of medical tourism destinations is the availability of health services. It is feasible for healthcare facilities with good human resources, medical equipment and service protocols to initiate activities related to medical tourism. The field of medical tourism is seeing rapid growth, prompting numerous governments to develop legal and practical strategies to accommodate this trend. Several factors contribute to the preference for international travel for medical purposes, including the reduction in transportation costs, increased income levels, the transfer of knowledge and technology, and the availability of competitive rates. Collaboration with health examination facilities is a frequent practice to establish, manage, assess, and improve customer relationship systems in the medical tourism industry (10, 11). This relationship marketing approach is essential to gain a competitive edge in the market.

This study aimed to investigate the literature on healthcare facilities and medical tourism from various countries that have established medical tourism sectors and analyse the influence of such facilities on medical tourism. The results of this literature review allow implications to be drawn concerning the significance of healthcare facilities for medical tourism worldwide.

## Method

**Figure 1:**
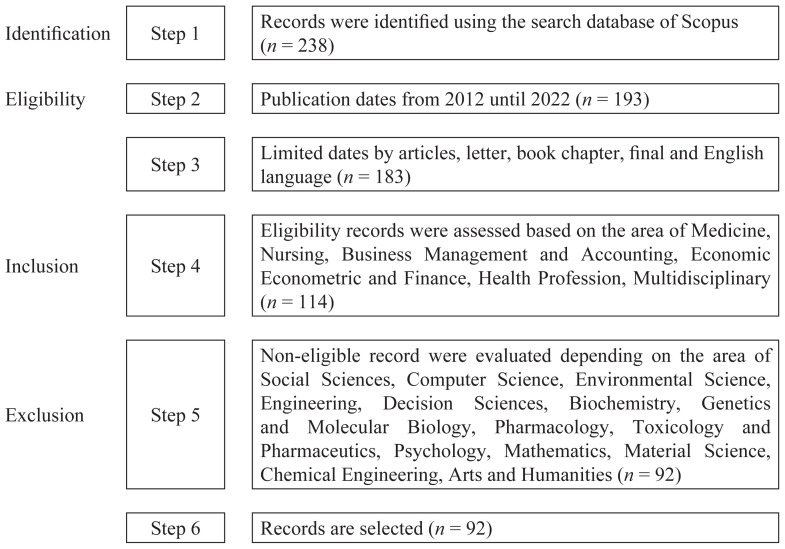
Steps in selecting articles

A detailed review of the literature was carried out. The Scopus database (https://www.scopus.com) was used to gather data from 2012–2022 to perform a bibliometric analysis (Figure 1). This analysis provided a global overview of the effect of healthcare facilities on medical tourism for the period in question. The number of research publications is one piece of data that can be obtained through this bibliometric analysis. Publications that use knowledge mapping examine medical-tourism-related healthcare facilities across the world. This use is integral to technology management; it encompasses various aspects, such as defining research programmes, making decisions about technology-related activities, designing knowledge-based structures, and formulating decisions regarding education and training. In connection with this, a landscape map is created, and science-related topics appear on it. Bibliographic information, keywords, quotations and other elements are used as input. Science mapping is a method utilised to visually represent a certain scientific domain, and it can be accomplished by performing bibliometric analysis (12). Research publications that map knowledge to analyse healthcare facilities used for medical tourism worldwide are employed to manage technology. This includes defining research programmes, determining technology-related activities, designing structures for knowledge bases, and deciding about education and training.

The context of healthcare medical tourism was based on the title or author keyword : ( ( TITLE-ABS-KEY ( Facility AND of AND healthcare ) AND TITLE-ABS-KEY ( health AND tourism ) ) ) AND ( LIMIT-TO ( SUBJAREA , “MEDI” ) OR LIMIT-TO ( SUBJAREA , “NURS” ) OR LIMIT-TO ( SUBJAREA , “BUSI” ) OR LIMIT-TO ( SUBJAREA , “ECON” ) OR LIMIT-TO ( SUBJAREA , “HEAL” ) OR LIMIT-TO ( SUBJAREA , “MULT” ) ) AND ( LIMIT-TO ( PUBYEAR , 2022 ) OR LIMIT-TO ( PUBYEAR , 2021 ) OR LIMIT-TO ( PUBYEAR , 2020 ) OR LIMIT-TO ( PUBYEAR , 2019 ) OR LIMIT-TO ( PUBYEAR , 2018 ) OR LIMIT-TO ( PUBYEAR , 2017 ) OR LIMIT-TO ( PUBYEAR , 2016 ) OR LIMIT-TO ( PUBYEAR , 2015 ) OR LIMIT-TO ( PUBYEAR , 2014 ) OR LIMIT-TO ( PUBYEAR , 2013 ) OR LIMIT-TO ( PUBYEAR , 2012 ) ) AND ( LIMIT-TO ( LANGUAGE , “English” ) ) AND ( LIMIT-TO ( EXACTKEYWORD , “Medical Tourism” ) ).

The Research Information Systems (RIS) file format was utilised to distribute the research map data. The bibliometric dominance map was developed based on analysising the Scopus menu search results and with VOSviewer and NVivo 12 Plus. The present study employed a descriptive methodology to assess the outcomes of a Scopus search by focusing on variables such as publication year, place of publication (nation) and research topic. During the interim period, a bibliometric map of research expansion in medical tourism and healthcare facilities was generated with VOSviewer. The gathered data was refined several times to generate the most accurate information about medical tourism and healthcare establishments. NVivo 12 Plus examined the interplay between the existing research’s indicators, variables and keywords. This study used bibliometric analysis to investigate the correlation between healthcare facilities and the phenomenon of medical tourism. Using bibliometric analysis at the undergraduate level allows for assessing academic publications by examining journal citations, authorship and other relevant metadata. This approach enables the evaluation of the effectiveness and efficiency of these scholarly works. Citations establish the relationships between an author and several elements, including subject matter, topic, methodology and other authors (13). Researchers can employ citation analysis for various purposes, such as mapping study areas to analyse their intellectual structures, evaluating academic implications and information sources, tracking the flow of ideas and knowledge, assisting with information retrieval, organisation and representation, and investigating academic literature’s users and usage patterns.

## Results

### Publications by Year

**Figure 2:**
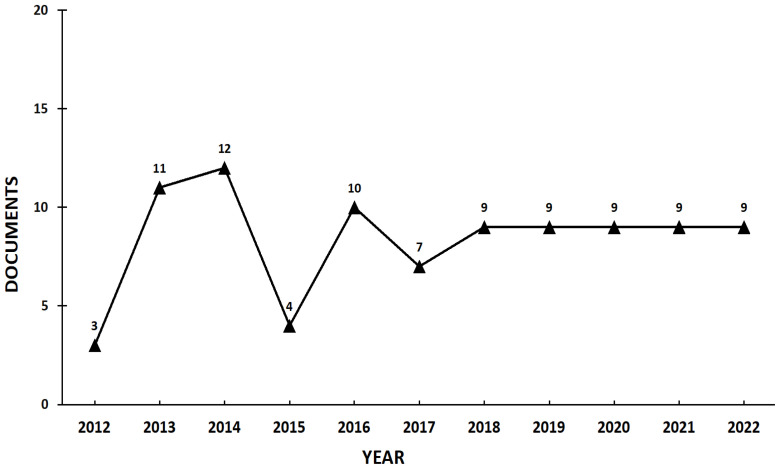
Number of publications by year of healthcare facility in medical tourism

Figure 2 shows the number of publications by year of healthcare facilities in medical tourism. The number of studies published in 2012 was three, the smallest in the last 10 years. In 2013, 11 articles were produced. In 2014, 12 papers were published, the highest number in the 10-year period examined here. In 2015, four articles were produced, which represents a considerable decrease compared to the previous year. In 2016, 10 studies were published; in 2017, the number was seven. From 2018 to 2022, only nine papers were produced in the last 5 years, which signals stagnation in the field.

### Publications by Country

**Figure 3:**
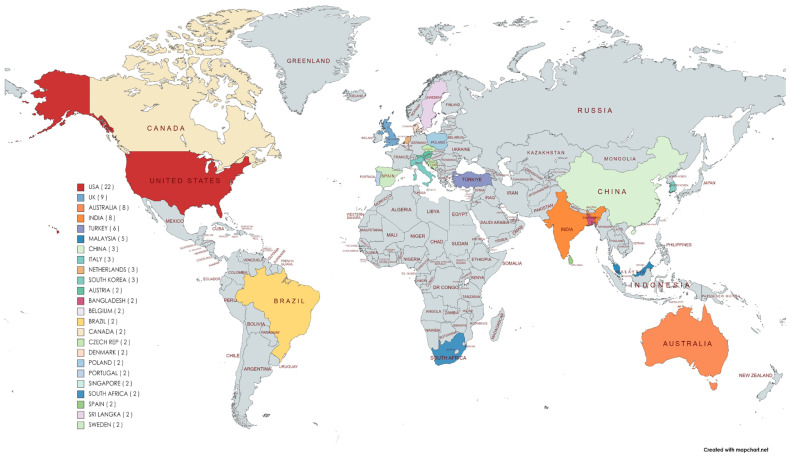
Scientific productions by country of a review healthcare facility in medical tourism

Twenty-four nations have produced studies on medical tourism and healthcare facilities. The United States is the country that has made the most significant contribution to the literature. The United States, England, Australia, India and Turkey are the top five nations regarding the number of literature reviews (Figure 3).

**Figure 4:**
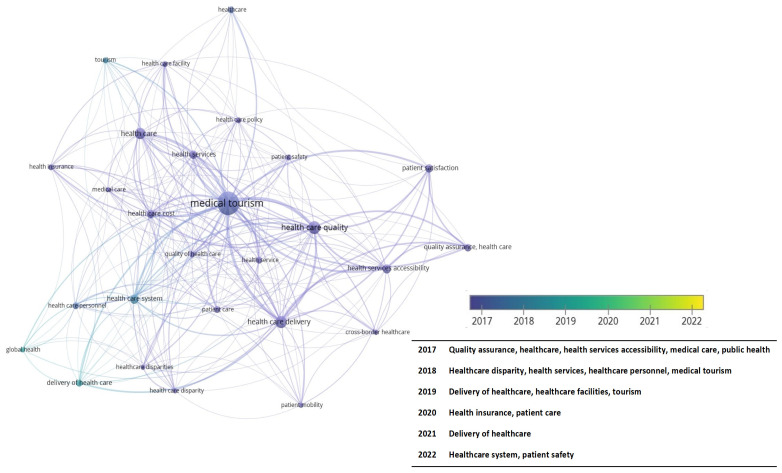
The keywords trends of a review healthcare facilities on medical tourism worldwide by year

Figure 4 shows the trending keywords for healthcare facilities and medical tourism that have existed for more than 5 years. Several studies have focused on patient safety and care in the past 5 years. During the same period, research on patient safety trends in healthcare facilities has not been conducted extensively.

As shown in Table 1, the keywords were discovered by utilising NVivo 12 Plus. As indicated by the graph, the percentages for the keywords ‘health,’ ‘medical,’ 'tourist,' 'care,' ‘facility’ and ‘healthcare’ were high.

### Overlay Visualisation of Titles and Abstracts

This study analysed how the relevant research patterns evolved through time. The trends observed in the medical tourism and healthcare facilities literature can be displayed to reflect an overall picture. Therefore, an overlay visualisation was also created with the analytical findings and the metadata imported into VOSviewer (14). In this representation, the node’s colour indicates the keyword and the year the article was published. The node’s darker hue signals the length of the topic’s discussion in the study. The graph in Figure 4 demonstrates how scholarly discussions addressed healthcare facilities and medical tourism from 2012 to 2022. The following graph illustrates which themes were most frequently discussed in that period, including medical tourism worldwide and healthcare facilities.

### Linking and Clustering the Themes and Abstracts Concerning Healthcare Facilities and Medical Tourism Worldwide

**Figure 5:**
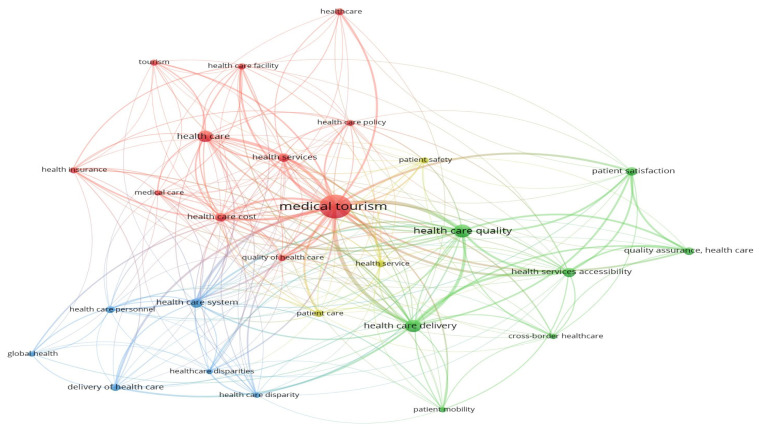
Network visualisation by keywords using VOSviewer of healthcare facilities in medical tourism

The concepts found in the 92 articles and various visualisations relating to this study area are described in Figure 5. This figure shows the four concept groups discovered during the review with VOSviewer. A list of concepts from each cluster is shown with a colour code. The objective is to identify the many recurring themes that have been extensively explored in previous studies and that can be effectively employed in future research. Figure 5 demonstrates how each cluster’s density can be distinguished using a different colour. The colours allow us to distinguish between the four clusters that are presented visually. Red is used for the medical tourism cluster, green for the healthcare quality, yellow for the health service and blue for the healthcare system. This visualisation makes it possible to identify different clusters within the topic being discussed. The cluster themes on medical tourism are shown in Table 2.

The visualisation demonstrates that the relationship between medical tourism and healthcare facilities has not been extensively examined; therefore, future research should focus on this topic.

## Discussion

Since 2012, few studies have been conducted on the effect of healthcare facilities on medical tourism in various countries. While the medical tourism industry is expanding, scholars have not investigated many factors influencing it. Hospitals’ healthcare facilities are one of the determining factors in attracting patients from abroad (15). Healthcare providers, including hospitals, clinics, and other service providers from various countries, increasingly focus on attracting international patients through direct online marketing and intermediaries such as facilitators (16, 17). The amount of research carried out has decreased recently. A literature review was performed to determine the role of health service facilities in developing medical tourism worldwide. Three articles were published in 2012 but increased in 2013 and 2014. This coincided with a rise in programmes aimed at developing tourism and the number of individuals travelling for medical reasons. In the past 5 years, from 2018 to 2022, relevant research has stagnated. This creates an opportunity for academics to investigate the various factors that influence healthcare facilities in fostering the growth of medical tourism. There is also a potential for advancing research methodologies and techniques.

The emergence and development of medical tourism have led to establishing and growing healthcare facilities in this sector. Multiple studies have shown that expanding health service facilities has positively impacted the growth of medical tourism (9). Concerning the theme of healthcare quality, the development of medical tourism linked to healthcare facilities is evident; developing these facilities in peripheral regions also contributes to improving medical tourism services (18). Recent studies have reviewed numerous facts about the healthcare system, which explains the theme of the same name. Several researchers have tried to understand how the healthcare system affects the growth of medical tourism. Healthcare system research has examined the cost-effectiveness and analytical accounting of medical tourism infrastructures and destinations (19). Many scholars have investigated the inefficiencies of healthcare systems and global health supply chains as part of the medical tourism phenomenon (20). The health service theme deals with patient care and safety. Appropriate communication and patient care are required for medical tourism to enhance the quality of healthcare for users (21).

The successful measures taken to develop this industry are evident in the significant increase in medical tourism exports to Asian countries in recent years (22). Thailand has emerged as a leading destination for medical tourism in recent years. The country’s medical tourism sector has grown significantly, with revenue reaching approximately USD340 million in 2010 and USD622 million in 2013 (23). This represents an average yearly growth rate of at least 20%. Several factors characterise the evolution of medical tourism in South Korea (24). The South Korean government has actively promoted medical tourism since revising legislation to allow domestic and private businesses to obtain medical services from outside the country. The government has developed 17 new growth industries in the top five categories. South Korea has made tourist-related health services a new development engine in its national strategy (24). Skilled health professionals and outstanding healthcare facilities will attract patients from abroad (25). The past few years have witnessed the rise of the global healthcare trend, in which healthcare mimics the internet in terms of the globalisation of knowledge and medical practice. This trend is in effect in many other industries and is a radically different way of approaching the healthcare market. Its driver is the search for lower costs. Still, many countries maintain a traditional stance, which results from decades of control by the usual players. This stance emphasises the professions with the most significant impact—doctors and nurses, their professional associations and insurance companies. The large number of patients who can travel worldwide looking for the best quality-price ratio for their healthcare has forced many health institutions to change their positions in the market (26).

Up to 83 publishers of scientific journals utilised the study’s findings regarding the impact of medical tourism on healthcare facilities. The *European Journal of Health Law* produces more articles than other journals. Due to the fact that its research is generated in the United States and many of its publishers are European health law journals, additional research is required. Over the past decade, articles have accounted for up to 75% of all global research projects, followed by reviews, papers, book chapters, correspondence and editorials. The United States published the most academic journals, followed by Great Britain, Australia, India and Turkey. Most research publications originated from these five countries, tourist destinations with exceptional health service facilities that encourage the growth of medical tourism (27, 28). More and more Americans are turning to medical tourism for cheaper medical care. It is not difficult to find countries that offer various procedures costing 30%–65% less than what is charged in the United States, given that the American healthcare system is the most expensive in the world.

The impact of healthcare facilities on medical tourism worldwide has not received sufficient scholarly attention. One of the requirements for offering medical tourism services of an adequate standard is the presence of infrastructure that paves the way for a growing number of patients to enjoy those services. Comprehensive and excellent healthcare facilities provide opportunities to expand medical tourism. A patient who is seeking medical treatment will undoubtedly choose a well-established facility. Thus, medical tourism will benefit significantly from healthcare facilities offering complete services to support diagnosis and therapy (27). Hospitals with good services and accompanying infrastructure will support medical tourism. Thus, the medical tourism industry encompasses more than just healthcare; for it to grow, many complementary support services must be available. These services are integral to the industry’s value chain (29).

Citizens from various nations, particularly developing countries with insufficient healthcare services, prefer to seek treatment with adequate healthcare facilities (25). This demonstrates the need for improved infrastructure and resources in healthcare systems to increase access to relevant services. The choice of medical tourism destination significantly impacts how health services are financed (30). Information on medical tourism services attracts people with low to intermediate incomes. When developing its medical tourism sector, Malaysia, an ASEAN member, transformed its health services by merging public and private providers. This policy gives the government primary, secondary and personal health services to provide tertiary healthcare (31). Thus, Malaysia’s private and public services are prepared to enhance existing healthcare facilities in the country’s medical tourism industry, which welcomes the highest proportion of Indonesian patients (32). Scholarly models of medical tourism describe the concept in depth by considering supply and demand (33). The service offered by a destination is referred to as the request. In the case of Malaysia, the government chose to attract medical tourists from Indonesia. Individual needs, evaluation services, medical facility services, the presence of relatives in the service destinations and the influence of medical service response bias towards treatment programmes are some elements that affect the choice of medical tourism destinations (31).

This study found that the topic of which healthcare facilities support medical tourism is still underexplored, and the demand for a strong healthcare system that can expand medical tourism still needs to be investigated. Quality healthcare facilities must be established to aid the healthcare system and promote the development of medical tourism. Furthermore, this study discussed the impact of healthcare facilities on the growth of medical tourism worldwide. Future research must perform a comparative analysis that involves the Scopus and Web of Science databases. This study has shortcomings that can be remedied using other online scientific resources.

## Conclusion

The following conclusion can be drawn from the above discussion. 2012–2022, most articles on medical tourism and healthcare facility development were published in 2018. Building on Heung’s (33) theory of the development of medical tourism, this study found that healthcare facilities have not been thoroughly investigated as components that play a role in expanding medical tourism, despite evidence suggesting this is the case. According to the findings, medical tourism was the most popular topic during the first 5 years of the examined period, but more research is needed on healthcare facilities. Medical tourism and, more recently, the healthcare system represented the main topics of the emerging research areas. Setting up a new facility in a healthcare system will foster the expansion of medical tourism.

## Figures and Tables

**Table 1:** Trending topics of keywords using NVivo 12 Plus

Word	Length	Count	Rate (%)
health	6	2,067	1.97
medical	7	2,007	1.91
tourism	7	1,788	1.70
care	4	873	0.83
facility	7	674	0.64
healthcare	10	642	0.61
international	13	527	0.50
patient	7	439	0.42
travel	6	431	0.41
service	7	403	0.38
management	10	402	0.38
services	8	386	0.37
study	5	305	0.29
public	6	261	0.25
patients	8	254	0.24
marketing	9	251	0.24
satisfaction	12	243	0.23

**Table 2:** Cluster’s themes on medical tourism

Clusters	Keywords	Top cite author
Cluster 1: Medical tourism	Medical tourism, health care, health care cost, health care facility, health care policy, health insurance, health service, healthcare, quality of health care, tourism	Medhekar A, Wong HY, Guiry M, Hall JE
Cluster 2: Health care quality	Health care delivery, health service accessibility, patient mobility, patient satisfaction, quality assurance, health	Iranmanesh M, Mathijsen A, Moghavvemi S, Mupara LM
Cluster 3: Healthcare system	Healthcare disparity, healthcare system, healthcare disparities, public health, global health, health care personal, delivery of health care	Spencer E, Tsoka-Gwegweni JM, Turner L, Zailani S, Abramson D
Cluster 4: Health service	Health service, patient care, patient safety	Adams K, Adler JT, Affleck Z, Aich A, Ajmera P, Al-Hashar A
